# Compound Heterozygous Structural Variants in Cases with Unsolved *PRKN* ‐Associated Parkinson's Disease

**DOI:** 10.1002/mds.70027

**Published:** 2025-08-30

**Authors:** Agata Fant, Sara Trova, Edoardo Monfrini, Gaia Treves, Francesco Musacchia, Fabio Landuzzi, Paola Mandich, Antonio Amoroso, Remo Sanges, Luca Pandolfini, Francesco Cavallieri, Franco Valzania, Valentina Fioravanti, Giulia Di Rauso, Gloria Brescia, Enza Maria Valente, Valeria Tiranti, Luigi Michele Romito, Chiara Reale, Barbara Garavaglia, Antonio Emanuele Elia, Andrea Cavalli, Alessio Di Fonzo, Manuela Vecchi, Stefano Gustincich

**Affiliations:** ^1^ Non‐Coding RNAs and RNA‐Based Therapeutics Italian Institute of Technology (IIT), CMP3VdA Aosta Italy; ^2^ Dino Ferrari Center, Neuroscience Section, Department of Pathophysiology and Transplantation University of Milan Milan Italy; ^3^ Foundation IRCCS Ca' Granda Ospedale Maggiore Policlinico, Neurology Unit Milan Italy; ^4^ Center for Human Technologies, Non‐Coding RNAs and RNA‐Based Therapeutics, Italian Institute of Technology (IIT) Genoa Italy; ^5^ Computational and Chemical Biology Italian Institute of Technology (IIT), CMP3VdA Aosta Italy; ^6^ Department of Neuroscience, Rehabilitation, Ophthalmology, Genetics and Maternal and Child Health University of Genoa Genoa Italy; ^7^ Area of Neuroscience, International School for Advanced Studies (SISSA) Trieste Italy; ^8^ Neurology Unit, Neuromotor & Rehabilitation Department Azienda USL‐IRCCS di Reggio Emilia Reggio Emilia Italy; ^9^ Clinical and Experimental Medicine PhD Program University of Modena and Reggio Emilia Modena Italy; ^10^ Department of Pathophysiology and Transplantation University of Milan Milan Italy; ^11^ Department of Molecular Medicine University of Pavia Pavia Italy; ^12^ Neurogenetics Research Centre, IRCCS Mondino Foundation Pavia Italy; ^13^ Medical Genetics and Neurogenetics Unit Fondazione IRCCS Istituto Neurologico Carlo Besta Milan Italy; ^14^ Department of Clinical Neurosciences, Parkinson and Movement Disorders Unit Fondazione IRCCS Istituto Neurologico Carlo Besta Milan Italy; ^15^ Center for Human Technologies, Computational and Chemical Biology, Italian Institute of Technology (IIT) Genoa Italy; ^16^ Centre Européen de Calcul Atomique et Moléculaire (CECAM), Ecole Polytechnique Fédérale de Lausanne Lausanne Switzerland

**Keywords:** PRKN, Parkinson's disease, WGS, structural variants, diagnostic

## Abstract

**Background:**

Biallelic mutations in the *PRKN* gene are a common cause of early‐onset Parkinson's disease (EOPD). In addition to single nucleotide variants, structural variants contribute substantially to the mutational profile of *PRKN*. A significant portion of patients with EOPD remains genetically unsolved.

**Objectives:**

By using short‐read whole genome sequencing (sr‐WGS), we aimed to uncover complex genetic alterations at the *PRKN* locus in EOPD cases which tested negative for mutations in Mendelian PD genes with clinical exome sequencing (CES) and multiplex ligation‐dependent probe amplification (MLPA).

**Methods:**

We evaluated 498 unrelated EOPD patients, who tested negative using gold‐standard diagnostic methods, using sr‐WGS. In selected cases, long‐read whole genome sequencing (lr‐WGS) with Oxford Nanopore technology was employed for an in‐depth analysis and validation. The Parkinson's Progression Markers Initiative (PPMI) dataset was interrogated to assess the prevalence of any newly identified elusive pathogenic genetic configurations.

**Results:**

sr‐WGS revealed elusive compound heterozygous structural variations, consisting of partially overlapping deletions and duplications within the *PRKN* gene in three unrelated EOPD cases (two familial, one sporadic). In familial cases, biallelic *PRKN* structural variants co‐segregated with the disease. The exact structure of each variant was resolved using lr‐WGS. Similar variants were absent in the large PPMI database, suggesting that they are a rare occurrence.

**Conclusions:**

In this article we describe a rare configuration of compound heterozygous structural variations involving partially overlapping chromosomal regions at the *PRKN* locus, which are difficult to detect through standard diagnostic genetic technologies. This study highlights the importance of integrating WGS into clinical practice. © 2025 The Author(s). *Movement Disorders* published by Wiley Periodicals LLC on behalf of International Parkinson and Movement Disorder Society.

Parkinson's disease (PD) is the second most common neurodegenerative disorder, affecting 1% of the population over the age of 60 years, with prevalence increasing to 4% by age 80 years.^1^ It is considered a multifactorial disorder, as both genetic and environmental factors can contribute to its pathology.^2^ While most cases are sporadic, up to 20% present as familial.^3^, ^4^ Variants in the *PRKN* gene are the most common cause of autosomal recessive PD, accounting for between 2.6% and 14.9% of early‐onset Parkinson's disease (EOPD) cases.^5^, ^6^, ^7^ Patients with *PRKN* mutations typically exhibit a median age of onset at 31 years, with a predominantly motor‐related presentation that responds well to dopaminergic therapy, progresses slowly, and shows little to no cognitive decline.^8^

The *PRKN* gene spans 1.3 Mb and contains 12 exons encoding for the 465 amino acid Parkin protein. *PRKN* is located within FRA6E, a common fragile site prone to genomic instability. Its core region, spanning exons 3 to 8, represents a known mutation hotspot, making it particularly susceptible to rearrangements.^9^ Parkin plays a crucial role in the physiology of dopaminergic neurons of the mesencephalon, the cell type that degenerates in PD. It is an E3 ubiquitin ligase and mediates mitophagy, which is the selective degradation of mitochondria, a function essential for mitochondrial homeostasis.^10^ Given its neuroprotective role, *PRKN* mutations compromise the ability of this protein to regulate mitochondrial function leading to dopaminergic cells degeneration and PD.^11^

The up‐to‐date typical diagnostic approaches in PD involve multiplex ligation‐dependent probe amplification (MLPA) and targeted next‐generation sequencing (NGS) gene panels or clinical/whole exome sequencing (CES/WES). However, these tests exhibit low sensitivity for complex structural variations. By taking advantage of these and other technologies, various types and combinations of *PRKN* pathogenic variants have been found to be causally associated to PD, including single nucleotide variants (SNVs) and structural variants (SVs).^8^ Nevertheless, an important portion of typical EOPD cases remain genetically unsolved.

In this study, we demonstrate that whole genome sequencing (WGS) can successfully solve some of these typical EOPD cases by identifying elusive combinations of SVs in the *PRKN* gene. Prior gold‐standard diagnostic methods had failed to detect these instances of compound heterozygosity, leaving these cases undiagnosed for years despite extensive genetic investigations.

## Methods

### Study Participants

Patients with PD, diagnosed according to International Parkinson and Movement Disorder Society (MDS) clinical diagnostic criteria,^12^ were enrolled in a prospective observational study, entitled NeurOmics (Prot. NeurOmics: 209/2024 ‐ DB id 13 896) designed to investigate omics alterations for the identification of pathogenic mechanisms and biomarkers in neurodegenerative disorders. Written informed consent for publication of pseudonymized genetic and clinical details was obtained from all the subjects involved. For this study cohort, 498 unrelated EOPD were selected by neurologists with expertise in movement disorders and neurogenetics to be negative or with inconclusive results to previous gold‐standard diagnostic assays (CES and MLPA) for genes known to be associated with Mendelian forms of PD (namely *SNCA*, *LRRK2*, *PRKN*, *PINK1*, *PARK7*, *DNAJC6*, *SYNJ1*, *VPS13C*, *PLA2G6*, *ATP13A2*, *FBXO7*, *RAB39B*, *PTRHD1*, and *GRN*). For diagnostic genetic testing in the clinical setting, CES was performed using the Sure Select CD Clinical Focused Exome kit (Agilent Technologies) that enables analysis of only disease‐associated targets (5629 targets in total) on the Illumina NextSeq2000 platform, and CNVs in *PRKN*, *PARK7, PINK1*, *SNCA*, and *ATP13A2* were analyzed by MLPA with the SALSA MLPA Parkinson probemix P051 (MRC Holland).

This cohort includes patients carrying monoallelic pathogenic variants in *PRKN* (n = 32) and *PINK1* (n = 2). Concerning the index cases of Families A and B, we enrolled additional family members, specifically n = 2 affected siblings and n = 4 first‐degree healthy relatives from Family A, and n = 1 affected and n = 1 healthy sibling from Family B. Clinical features of the whole cohort are reported in Table S1.

### DNA Extraction and Quality Assessment

Genomic DNA was extracted from 400 μL of fresh whole blood using the Qiacube Automated Nucleic Acid Extraction Kit on the Qiacube Connect instrument (Qiagen), following the manufacturer's instructions.

The extracted genomic DNA (gDNA) was quantified using a Nanodrop OneC spectrophotometer (Life Technologies, Thermo Fisher Scientific) to assess purity ratios, and a Qubit Fluorometer 4.0 (Thermo Fisher Scientific) to determine nucleic acid concentration (ng/μL) using the Qubit™ dsDNA High Sensitivity Assay Kit (Invitrogen™, Thermo Fisher Scientific). The quality of gDNA was also assessed for size distribution using the 4200 TapeStation system (Agilent Technologies) with the Genomic DNA ScreenTape. For all samples, the DNA integrity number (DIN) was >7 as required for Nanopore sequencing.

### Short‐Read Whole Genome Sequencing

sr‐WGS of gDNA extracted from blood samples was performed with the Illumina NovaSeq 6000 platform (Illumina Inc.) to produce paired 151 base pair reads. The library preparation and sequencing of gDNA from the blood samples utilized barcode adapters. Specifically, 350 ng of gDNA was prepared using the Illumina DNA PCR‐Free Prep, Tagmentation library preparation kit (Cat. No. 20041795), and IDT® for Illumina® DNA/RNA Unique Dual Indexes Set A and B, Tagmentation (96 indexes, Cat. Nos 20027213 and 20027214). The library preparation was carried out with a T100 Thermal Cycler instrument (Bio‐Rad) and standard laboratory equipment. Libraries were pooled by volume and adjusted for the index correction factor according to Illumina's instructions to ensure balanced sample coverage across each run. An average coverage of 39.1X (±8.7) was achieved for Families A and B and the single case (Table S2).

Alignment on GRCh38 and variant calling for SNVs was performed with the GPU optimized version of the Genome Analysis Toolkit (GATK) developed by NVIDIA Clara™ Parabricks®. For SV detection, to decrease false‐positive calls, we use four different tools (Manta v1.6.0,^13^ Lumpy v0.2.13,^14^ CNVnator v0.3.3,^15^ and BreakDancer v1.4.5^16^) making them a consensus using Survivor (v1.0.7)^17^ and selecting only SVs detected by at least two tools. SVs were annotated first with SVAfotate^18^ to obtain population level allele frequency information and then with AnnotSV v3.2.3.^19^

### Long‐Read Whole Genome Sequencing

Selected gDNA samples used for sr‐WGS were also analyzed by long‐read whole genome sequencing (lr‐WGS). The gDNA was further fragmented by pipetting it through a p20 tip (Gilson) and fragments below 10 kb were removed using the Short Read Eliminator XS (SRE XS, PacBio® #102‐208‐200). Library preparation was performed using the DNA Ligation Sequencing Kit (SQK‐LSK114) according to the manufacturer's instructions. Long‐read sequencing was performed on a FLO‐PRO00114M flow cell (R10.4.1) using the PromethION 24 sequencer (Oxford Nanopore Technologies, ONT). For all samples, the starting material was >1500 ng and a total of >500 ng was obtained for each library. Initially, 30–50fM were directly loaded on the flow cell and after 36 hr a second loading with 30–50fM was required, following a flow cell nuclease wash (according to ONT instructions). An average coverage of 23.6X (±6.9) and average N50 of 22.5 kb (±5.9) was achieved for Families A and B and the single case (Table S3).

Basecalling and concurrent genome alignment were performed with dorado (ONT; v0.6.0). For variant calling and phasing, the ONT wf‐human‐variation pipeline v.2.2.4 was used, with options “–sv –snv –cnv –phase”. Sniffles2 options were modified with “–long‐del‐length 520000 –long‐dup‐length 520000” to increase the SV size limits of detection.

### Validation of Compound Heterozygous SVs in *PRKN* Using PCR

The same gDNA used for the WGS analysis was used for the validation with polymerase chain reaction (PCR). Additional gDNA for PCR validation was available for I‐2, II‐1, II‐2, II‐3 in Family A, for II‐1 and II‐2 in Family B, and for the single case (SC).

The primers for each SV were designed with the PRIMER BLAST tool^20^ from the National Center for Biotechnology Information (NCBI) website to target the regions surrounding the breakpoints of the deletion and duplication (Fig. S1), after checking for non‐specific regions of annealing with the same online tool. The PCR reaction was carried out with TaKaRa Ex Taq DNA Polymerase according to manufacturer's instructions in a T100 Thermal Cycler (Biorad) under the following conditions: 30 cycles of 98°C for 10 s, 60°C for 30 s, 72°C for the time needed for the elongation (60 s each 1000 bp amplified). Around 50 ng of DNA were used as input for each sample. All samples were run in a 2% agarose gel made with 1X TAE buffer and SYBR Safe DNA Gel Stain (Invitrogen), and GeneRuler 100 bp Plus DNA (Invitrogen) was used as ladder. Commercial primers for glyceraldehyde 3‐phosphate dehydrogenase (GAPDH) were used as the positive control (Hs02786624_g1, Thermo Fisher). All the primers were newly designed (Table S4).

### Analysis of WGS Data of the PPMI Cohort

Data used for this article were obtained on November 15, 2020 from the Parkinson's Progression Markers Initiative (PPMI) database (www.ppmi-info.org/access-dataspecimens/download-data), RRID:SCR_006431. Clinical information for the reanalyzed cases was obtained from the curated data provided by the PPMI data team in the latest PPMI release (21‐03‐2025) and is summarized in Table S5. A total of 851 subjects were included, all lacking pathogenic variants in *LRRK2*, *PINK1*, *PRKN*, or *SNCA* according to the curated PPMI data. Information about pathogenic variants was obtained from Participant_Status and iu_genetic_consensus tables. This cohort comprised 144 prodromal individuals, 63 SWEDD cases (clinically diagnosed with PD but with normal dopamine transporter single photon emission computed tomography [DAT SPECT]), 195 healthy individuals, and 449 PD patients (87 EOPD, 354 late‐onset Parkinson's disease [LOPD], and 8 without available onset information). SV detection was performed using the same pipeline as previously described.

## Results

### Study Cohort

Our study cohort comprised 498 unrelated typical EOPD patients from Italy. In addition, three affected and five healthy relatives from two families were enrolled. The larger family, designated as Family A, consists of two healthy parents and five siblings (three affected), while the other family, Family B, is composed of three siblings (two affected). Clinical and demographic features of the analyzed cohort are reported in Table S1. All EOPD cases were previously analyzed using gold‐standard diagnostic assays (CES and MLPA) yielding negative or inconclusive results for genes known to be associated with Mendelian forms of PD (see Methods).

### Identification of Biallelic SVs in *PRKN* by WGS

**FIG. 1:**
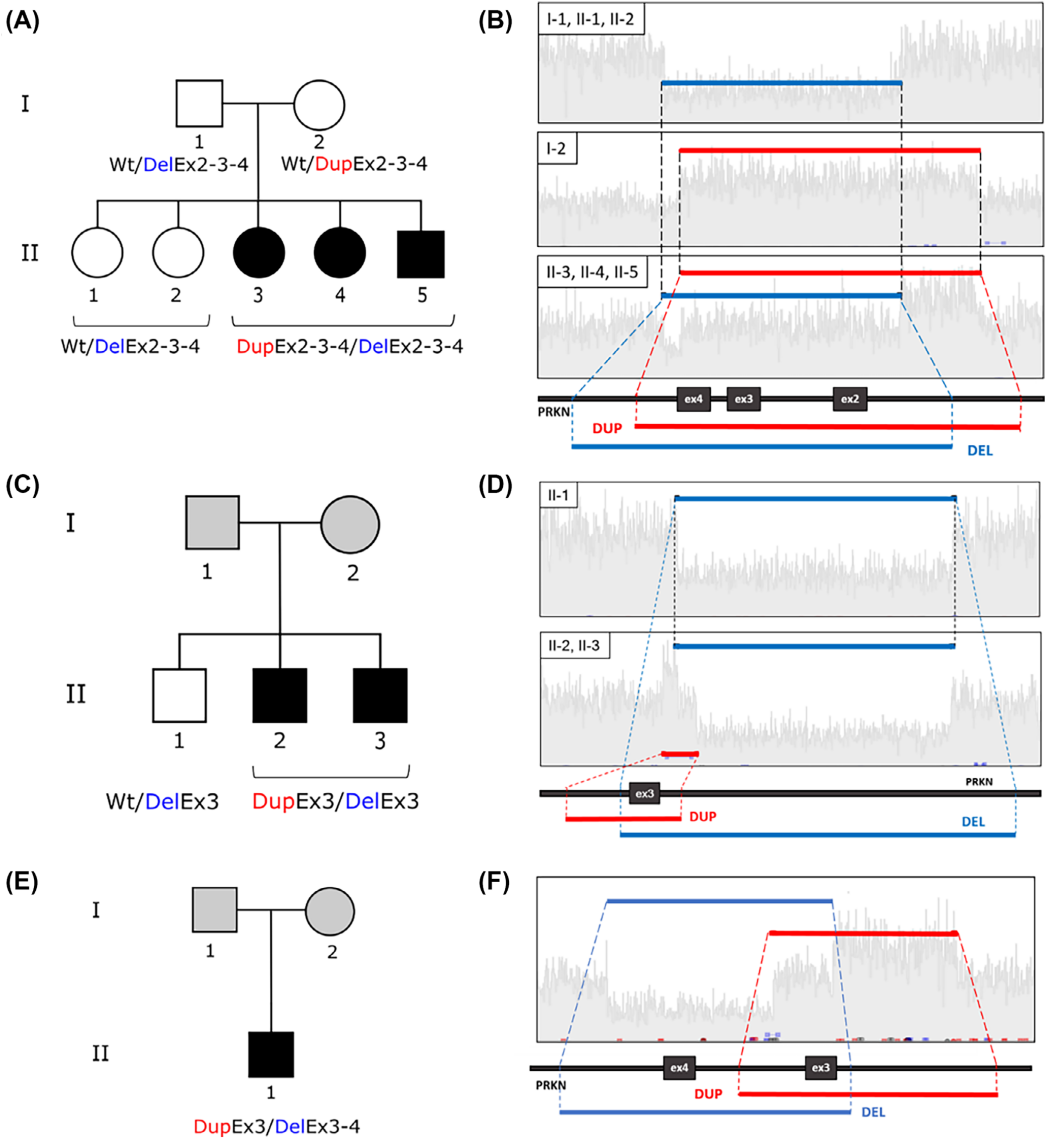
Configuration of compound heterozygous structural variations of *PRKN* involving partially overlapped regions. (A, C, E) Pedigrees of Family A, Family B, and the single case, respectively, showing affected individuals (black symbols) and subjects not sequenced (grey symbols). (B, D, F) Samplot images depicting the deletions and duplications in *PRKN* identified in Family A, Family B, and the sporadic case, respectively. [Color figure can be viewed at wileyonlinelibrary.com]

sr‐WGS was employed to reconstruct the *PRKN* gene locus. To analyze WGS data, we implemented an ad hoc GPU‐based pipeline for SNV variant calling and a consensus approach of four different tools (Manta v1.6.0,^13^ Lumpy v0.2.13,^14^ CNVnator v0.3.3,^15^ and BreakDancer v1.4.5^16^) using Survivor (v1.0.7)^17^ to detect SVs (as described in Methods). We initially analyzed the two unrelated probands of Family A and Family B and identified a peculiar configuration of compound heterozygous SVs of *PRKN* involving partially overlapping regions. For these two familial cases, we extended the analysis to available family members (Fig. 1A,D).

Regarding Family A, genomic DNA was available for all the members of the family, namely two healthy parents, two healthy sisters, and three affected siblings. Bioinformatic analysis identified in the three affected individuals (II‐3, II‐4, and II‐5) a unique combination of SVs in the *PRKN* gene, consisting of a partially overlapping duplication (of ~509 kb) and a deletion (of ~405 kb) in *trans*, both spanning exons 2–3–4 of *PRKN* (Fig. 1A,B, Table 1). The two healthy sisters (II‐1, II‐2) carried only the deletion, inherited from the father (I‐1), while the duplication present in the three affected siblings was inherited from the mother (I‐2) (Fig. 1A,B).

**Table 1:** Location of the breakpoints of all the PRKN structural variants identified in the two families and the sporadic Parkinson's disease case with short‐read whole genome sequencing, aligned on hg38.

Family	Subjects	Location	Start	End	SV type	Size (bp)	Exons involved
A	I‐1, II‐1, II‐2, II‐3, II‐4, II‐5	6q26	162,154,938	162,560,715	Del	405,964	2,3,4
	I‐2, II‐3, II‐4, II‐5		162,185,434	162,695,422	Dup	508,975	2,3,4
B	II‐1, II‐2, II‐3	6q26	162,260,786	162,393,577	Del	133,194	3
	II‐2, II‐3		162,253,473	162,269,839	Dup	15,125	3
Single case	SC	6q26	162,149,134	162,265,728	Del	116,594	3,4
			162,234,822	162,328,795	Dup	93,973	3

Abbreviations: SV, structural variant; bp, base pair; del, deletion; dup, duplication.

In Family B, genomic DNA was available for two affected and one healthy brother. Both affected siblings (II‐2, II‐3) carried a ~15 kb duplication and a ~133 kb deletion both spanning exon 3 of *PRKN* while the healthy brother (II‐1) presented the deletion only (Fig. 1C,D; Table 1).

Collectively, we demonstrated the biallelic configuration of the identified *PRKN* SVs (Fig. 1).

We then extended the sr‐WGS analysis to the entire EOPD cohort. An additional sporadic EOPD case (SC) was detected carrying a *PRKN* deletion involving exons 3 and 4 on one allele, and a duplication of exon 3 on the other allele (Fig. 1E,F; Table 1). This configuration was not captured by MLPA, as only a dosage alteration of exon 4 was detected (Fig. S2).

WGS did not identify any relevant variants in other PD genes in these carriers (see Methods).

All the identified SVs are classified as pathogenic (https://www.mdsgene.org/d/1/g/4?fc=0&mu=0&_mu=1&_country=1) and their association in *trans* indicated that *PRKN* variants were the genetic cause of EOPD in these cases. The presence of overlapping deletions and duplications with breakpoints in introns in the two different alleles of *PRKN* explains the missed diagnosis through gold‐standard diagnostic methods, as MLPA could not reveal exon dosage abnormalities and CES did not cover these regions (Fig. S2). Overall, three unrelated EOPD cases (0.6%, 3/498), two familial and one sporadic, were found to carry a peculiar configuration of compound heterozygous SVs of *PRKN* involving partially overlapping regions. The clinical phenotype of carriers was highly suggestive of *PRKN*‐related PD (Table 2).

**Table 2:** Clinical information of the affected Parkinson's disease cases with compound heterozygous PRKN structural variants.

Parameter	Family 1			Family 2		Proband 3
	M	F	F	M	M	M
Age at onset (years)	32	40	34	38	37	40
Disease duration (years)	19	14	6	14	13	22
Non‐motor symptoms	Sleeplessness, RLS	Anxiety, constipation	None	Constipation	Anxiety, depression, constipation	Depression, ICD (hypersexuality with dopamine‐agonist)
Motor symptoms at onset	Right‐hand rest tremor	Bilateral lower limbs bradykinesia	Right leg rest tremor	Left foot dystonia	Rigidity	Right upper limb bradykinesia
Other motor symptoms	Bradykinesia, rigidity.	Bradykinesia, rigidity, mild for limbs and gait ataxia.	Bradykinesia, rigidity	Bradykinesia, rigidity	Bradykinesia	Tremor
Clinical improvement of motor symptoms with levodopa	Yes	Yes	Yes	Yes	Yes	Yes
Current LEDD (mg)	105	790	105	660	300	900
Deep brain stimulation	No	No	No	No	No	Yes, favorable response (after 13 years of disease duration)
Other advanced therapies	No	No	No	No	No	No

Abbreviations: M, male; F, female; RLS, restless legs syndrome; ICD, impulse control disorder; LEDD, levodopa equivalent daily dose.

**FIG. 2:**
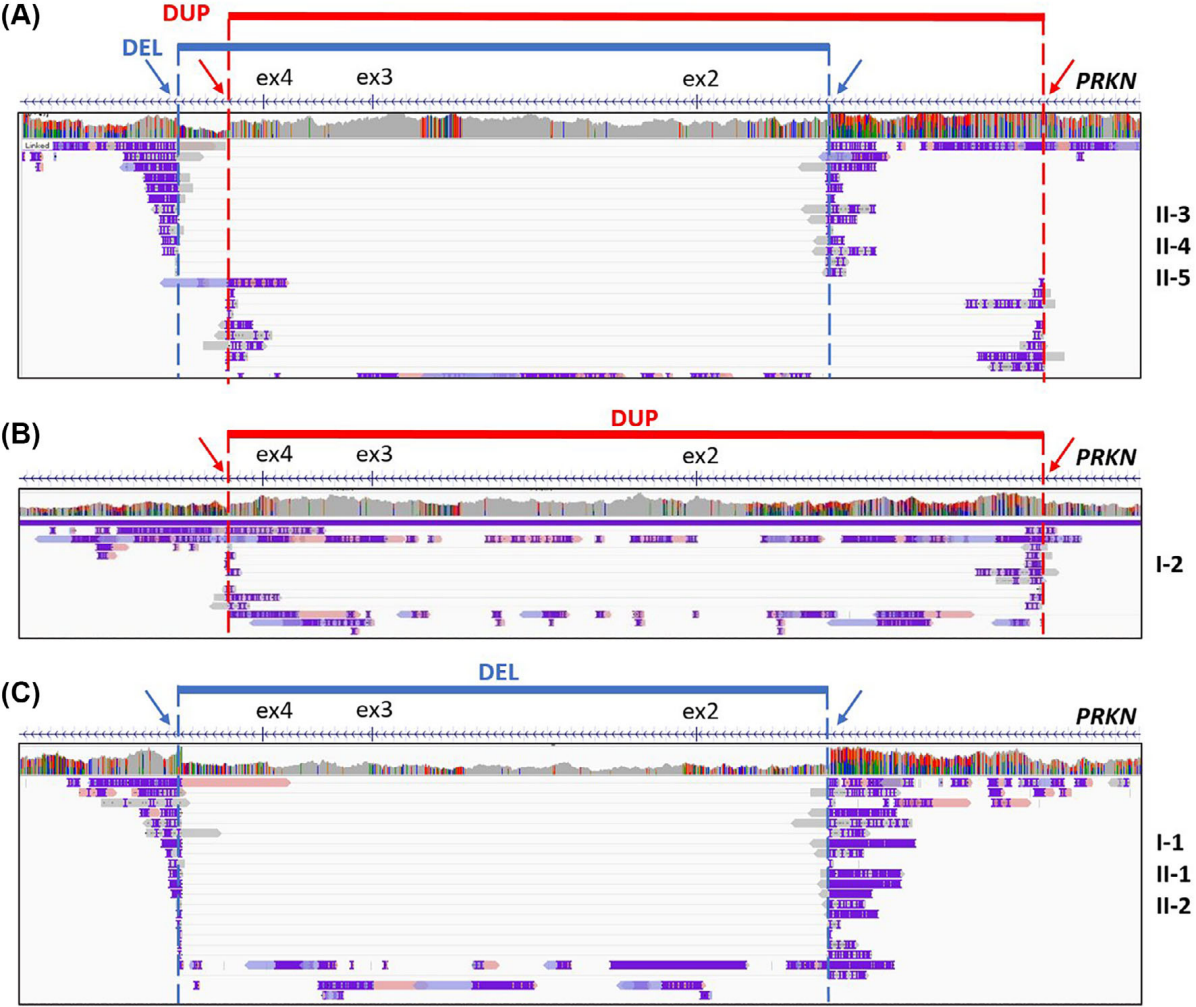
Analysis of the identified biallelic *PRKN* structural variants by long‐read sequencing in Family A. Representative images from the Integrative Genomics Viewer (IGV) of long‐read sequencing split reads mapped on the *PRKN* gene of Family A, displaying the deletion of exons 2–3‐4 (blue) and the duplication of exons 2–3‐4 (red) in all the three affected siblings (A), the duplication in the mother (B), and the deletion in the father and unaffected siblings (C). Blue and red arrows indicate deletion and duplication breakpoints, respectively. Gray reads indicate the default state for reads that match the reference genome. Pink and violet reads are supplementary alignments. [Color figure can be viewed at wileyonlinelibrary.com]

For a precise characterization of the compound heterozygous *PRKN* SVs identified by sr‐WGS, lr‐WGS was also performed in all the probands and available family members, taking advantage of the Oxford Nanopore Technologies platform. lr‐WGS confirmed the presence of biallelic *PRKN* SVs (Fig. 2; Fig. S3) in PD probands and monoallelic *PRKN*‐SVs in healthy relatives of the two analyzed families. Additionally, lr‐WGS allowed us to precisely determine all the SVs breakpoints, which were confirmed through genomic PCR amplification (Table 1; Table S4; Fig. S1).

### Analysis of Compound *PRKN* SVs in the PPMI Cohort

The analysis was extended to the WGS data extracted from PPMI dataset by applying the same tailored bioinformatic pipeline to detect SVs. We analyzed a total of 851 individuals without pathogenic variants in *LRRK2*, *PINK1*, *PRKN*, or *SNCA*, including 449 PD patients (87 EOPD, 354 LOPD, and 8 with unknown age at onset), 144 prodromal subjects, 63 SWEDD cases (clinically diagnosed with PD but exhibiting normal DAT SPECT imaging), and 195 healthy controls. The clinical characteristics of these groups are detailed in Table S5.

Initially, we compared the sequencing quality metrics between the PPMI and our Italian cohort and found no significant technical differences. Both cohorts were sequenced using Illumina technology with a PCR‐free library preparation kit, achieving comparable average coverage: 34.79X (± 4.96) in the PPMI cohort versus 34.02X (± 7.13) in this Italian cohort. When focusing specifically on the EOPD subsets, 87 samples from PPMI and 498 from our cohort, the average coverage remained similar at 35.48X (± 5.29) versus 34.02X (±7.1), respectively. No substantial technical or clinical differences were observed between the two cohorts (Table S1; Table S5), although the PPMI's EOPD subset was notably smaller than the cohort of the present study. Overall, 13 heterozygous SVs (either a deletion or a duplication comprising exons) were detected in the *PRKN* gene of all PPMI cohorts (Table S6). Nonetheless, no patient showed either compound SVs heterozygosity involving both a deletion and a duplication spanning overlapping genomic regions within the *PRKN* locus or compound heterozygosity with a pathogenic SV and a single nucleotide variant in *PRKN*.

## Discussion

Current technologies used in clinical practice are precious tools to detect pathogenic variants in PD‐related genes with high confidence and reproducibility.^21^, ^22^ However, they present important limitations. Accumulating evidence indicates that SNVs or SVs could remain undetected due to challenges in accurately mapping these variants within analyzed sequences and the inherent difficulties in identifying SVs without introducing artifacts.^23^, ^24^ This limitation is particularly relevant when clinical phenotypic traits suggest the involvement of a specific disease‐causing gene. The co‐existence of inheritance, early‐onset, and specific symptoms is typical of *PRKN*‐PD patients.^25^ Nevertheless, in several cases with a typical *PRKN*‐PD clinical presentation, no pathogenic *PRKN* variants are identified, even after years of molecular diagnostic testing using traditional approaches.^26^

In this study, we demonstrate that both short‐read and long‐read WGS approaches can accurately detect a rare configuration of compound heterozygous SVs in the *PRKN* gene (Figs. 1 and 2; Fig. S3). This configuration was identified in six Italian EOPD individuals, including five familial cases from two unrelated families and one sporadic patient, all of whom had previously undergone CES and MLPA analyses that were either negative or inconclusive (Fig. 1). All the identified variants involve partially overlapping chromosomal regions comprising exon 3, consistent with its location within FRA6E, a common fragile site known to be prone to genomic instability.^9^ This compound heterozygous SVs configuration differs from previously reported SV patterns, such as two deletions or two duplications in *trans* ^27^ or a duplication on one allele entirely overlapped by a deletion on the other, as recently reported in one European family and in one German EOPD case ^28^, ^29^.

Our data corroborate recent findings reporting biallelic complex alterations in the *PRKN* gene in unsolved cases carrying a single heterozygous *PRKN* mutation and further expand the genetic heterogeneity of this specific locus in EOPD patients ^30^. Additionally, we unveiled partially overlapping SVs in *PRKN* also in patients in which conventional methods did not show any pathogenic *PRKN* variant. These findings from the PD cohort enrolled in Italy underscores the broader relevance of complex *PRKN* SVs in EOPD across diverse ancestral backgrounds. Finally, we calculated the prevalence of this distinctive type of genetic alteration, occurring in 0.6% of EOPD patients who had previously tested negative using standard diagnostic approaches.

A comprehensive analysis of the PPMI study dataset did not reveal any elusive biallelic *PRKN* SVs event in 87 EOPD cases, suggesting that these events occur at low frequency. As the sample size of the PPMI EOPD subset is limited (approximately one‐sixth of the cohort presented in this study) we cannot exclude that the PPMI cohort lacked sufficient power to detect these rare SVs in *PRKN*. Of note, the PPMI cohort represents a more genetically and geographically diverse population than the relatively homogeneous Italian cohort included in the present study. The diversity in ethnic background may contribute to the variability in the detection of rare, population‐specific SVs in *PRKN*.

These elusive SVs were confirmed using a third‐generation sequencing platform, Oxford Nanopore Technologies (Fig. 2; Fig. S3). Nonetheless, sr‐WGS was sufficient to accurately detect these compound heterozygous SVs without the need of more advanced technologies (Fig. 1; Table 1). sr‐WGS effectively identifies the elusive biallelic SV configurations when analyzed with split read‐based methods. Since breakpoints are located within intronic regions, area typically not covered by CES and/or WES, these SVs had previously gone undetected. In contrast, WGS, with its uniform coverage of both exonic and intronic regions and the availability of specialized bioinformatics tools, significantly improves the detection of variants that escape conventional genetic approaches. Furthermore, these genomic variants would likely be missed by tools that rely exclusively on read depth or by quantitative methods such as MLPA. Although the adoption of WGS as a first‐line diagnostic approach still requires the implementation of appropriate technological and bioinformatics infrastructure in healthcare systems, its improved diagnostic yield and steadily decreasing cost support its use in selected patient populations.

Based on our findings, we propose the use of WGS to diagnose both familial and sporadic EOPD cases at large and, in particular, in those individuals that present clinical features suggestive of *PRKN*‐PD and have tested negative or inconclusive by conventional methods, including targeted gene panels, CES/WES, and MLPA. Rather than limiting this analysis to individuals carrying monoallelic mutation in autosomal recessive genes (eg, *PRKN*, *PINK1*, etc.), WGS should be considered for a broader group of unresolved PD cases.

The choice between sr‐WGS versus lr‐WGS as second‐line diagnostic test largely depends on the available computational infrastructure, laboratory equipment, and bioinformatic expertise within hospitals and specialized centers. sr‐WGS can efficiently detect these elusive compound heterozygous SVs and is less technically demanding than lr‐WGS. However, it may be less accurate for identifying large or complex rearrangements.^31^, ^32^ Therefore, a sequential strategy, starting with sr‐WGS and reserving lr‐WGS for cases that remain unresolved, may optimize diagnostic yield. For familial or sporadic EOPD cases that remain undiagnosed after MLPA/CES/WES and sr‐WGS, lr‐WGS may provide a definitive diagnosis. By integrating both sequencing approaches, the likelihood of uncovering the genetic cause in undiagnosed patients is significantly enhanced. Identifying a definitive genetic diagnosis can also inform clinical decision‐making and potential therapeutic strategies. As WGS costs decline, its application is expected to expand, particularly among patients with strong indications of a monogenic etiology. While WGS, both sr‐WGS and lr‐WGS, demonstrates improved diagnostic yield in PD, a formal cost‐effectiveness analysis compared with WES or traditional testing is warranted prior to its adoption as a first‐line diagnostic tool.

For the remaining unsolved EOPD cases in our cohort, we are currently performing an in‐depth analysis of WGS data by exploring repeat expansions and non‐coding regions to identify and characterize variants in key regulatory elements, such as enhancers and promoters, and to assess their potential impact on three‐dimensional genome organization. These investigations may require the integration of WGS with complementary datasets, including chromatin interactions maps (eg, Hi‐C) and epigenetic profiles (eg, histone modifications). Such integrated multi‐omics approaches offer a comprehensive framework for unraveling complex disease mechanisms, identifying potential biomarkers for diagnosis and prognosis, and advancing precision medicine in PD. Finally, given emerging evidence suggesting a potential role of somatic mutations in neurodegenerative diseases,^33^, ^34^, ^35^, ^36^ comparative genomic analysis between post‐mortem neuronal tissue and peripheral blood may yield novel insights into the molecular basis of unresolved cases and uncover pathogenetic mechanisms relevant for the development of new therapeutic strategies.

In conclusion, as genomic technologies become increasingly accessible, our findings strongly support the clinical implementation of sr‐WGS to uncover previously undetectable SVs. This approach promises to improve diagnostic accuracy and advance the application of precision medicine in PD.

## Author Roles

(1) Research Project: A. Conception, B. Organization, C. Execution; (2) Data Analysis: A. Design, B. Execution, C. Collection, D. Review and Critique; (3) Manuscript Preparation: A. Writing of the First Draft, B. Review and Critique; (4) Clinical Activity: A. Supervision and Organization, B. Data and Sample Collection.

A.F.: 1B, 1C, 2B, 2C, 2D, 3A, 3B.

S.T.: 1B, 1C, 2B, 2C, 2D, 3A, 3B.

E.M.: 1B, 1C, 2C, 2D, 3A, 4B.

G.T.: 1C, 2B, 2C, 3A, 3B.

F.M.: 2A, 2C, 3B.

F.L.: 2C, 3B.

P.M.: 2C, 2D, 3A, 3B, 4A, 4B.

A.A.: 3A, 3B.

R.S.: 2D, 3B.

L.P.: 2A, 2D, 3B.

F.C.: 3B, 4B.

F.V.: 3B, 4B.

V.F.: 3B, 4B.

G.D.R.: 3B, 4B.

G.B.: 3B, 4B.

E.M.V.: 3B, 4B.

V.T.: 3B, 4B.

L.M.R.: 3B, 4B.

C.R.: 3B, 4B.

B.G.: 3B, 4B.

A.E.E.: 3B, 4B.

A.C.: 2D, 3B.

A.D.F.: 1A, 1B, 2D, 3A, 3B, 4A.

M.V.: 1A, 1B, 1C, 2A, 2C, 2D, 3A, 3B.

S.G.: 1A, 1B, 2A, 2D, 3A, 3B.

## Full Financial Disclosure for the Previous 12 Months

A.D.F. received honoraria from Sanofi for expert meeting participation. None of the other authors report any conflicts of interest.

## Supporting information


**Fig. S1.** Validation of the identified compound heterozygous structural variations of PRKN by genomic polymerase chain reaction (PCR) analysis. Primers designed to detect deletions (blue) and duplications (red) in family A (A), family B (B), and the single case (C). Agarose gel electrophoresis of the PCR products around the breakpoints of the deletion (left part of the gel) and the duplication (right part of the gel) in individuals II3, II‐1, II‐2, and I‐2 of family A (D), in individuals II‐2 and II‐1 of family B (E), and in the single case (F). Amplicon sizes (base pairs, bp) are indicated; amplicon size for GAPDH control is 157 bp. PF, primer forward; PR, primer reverse; WT, wild‐type; DEL, deletion; DUP, duplication; Ctrl–, negative control; SC, single case.

**Fig. S2.** Multiple ligation‐dependent probe amplification (MLPA) analysis of a panel of Parkinson's disease (PD)‐associated genes, including PRKN. Representative images of MLPA analysis for one PD affected member of family A (A), of family B (B), and the single case (C).

**Fig. S3.** Analysis of the identified biallelic PRKN structural variants by long‐read sequencing in family B and the single case. Representative images from the Integrative Genomics Viewer (IGV) of long‐read sequencing split reads mapped on the PRKN gene showing both the deletion (blue) and the duplication (red) of exon 3 in the two affected siblings (A) and only the deletion (blue) of exon 3 in the healthy brother (B) of family B, and with the concomitant deletion (blue) of exon 3 and 4 and duplication (red) of exon 3 in the single case (C). Gray reads indicate default state for reads that match the reference genome. Pink and violet reads are supplementary alignments.

**Table S1.** Clinical and demographic characteristics of patients included in the study. The table summarizes sex, age at disease onset, ethnicity, and family history of the disease.

**Table S2.** Summary metrics of short‐read whole genome sequencing (sr‐WGS) data of the two family members and sporadic Parkinson's disease (PD) patient with compound heterozygous PRKN structural variants (SVs).

**Table S3.** Summary metrics of long‐read whole genome sequencing (lr‐WGS) data of the two family members and the single case with compound heterozygous PRKN structural variants (SVs).

**Table S4.** Primer sequences designed around the junction breakpoints for polymerase chain reaction (PCR) analysis.

**Table S5.** Clinical and demographic characteristics of Parkinson's Progression Markers Initiative (PPMI) patients included in the study. The table provides information on sex, age at disease onset, ethnicity, age at diagnosis, family history of the disease, and the presence of key motor symptoms.

**Table S6.** Deletions and duplications in PRKN identified in the Parkinson's Progression Markers Initiative (PPMI).

## Data Availability

Anonymized patient data will be made available on reasonable request. Data used in this study are available from the PPMI database (https://www.ppmi-info.org/access-data-specimens/download-data).
