# Supplementary material for: Compound Heterozygous Structural Variants in Cases with Unsolved PRKN ‐Associated Parkinson's Disease
Source: Mov Disord. 2025 Aug 30;40(12):2722–31. doi: 10.1002/mds.70027 (PMC12710201; doi:10.1002/mds.70027)
Supplement: Supplementary file 1 — Fig. S1. Validation of the identified compound heterozygous structural variations of PRKN by genomic polymerase chain reaction (PCR) analysis. Primers designed to detect deletions (blue) and duplications (red) in family A (A), family B (B), and the single case (C). Agarose gel electrophoresis of the PCR products around the breakpoints of the deletion (left part of the gel) and the duplication (right part of the gel) in individuals II3, II‐1, II‐2, and I‐2 of family A (D), in individuals II‐2 and II‐1 of family B (E), and in the single case (F). Amplicon sizes (base pairs, bp) are indicated; amplicon size for GAPDH control is 157 bp. PF, primer forward; PR, primer reverse; WT, wild‐type; DEL, deletion; DUP, duplication; Ctrl–, negative control; SC, single case. [file MDS-40-2722-s003.pdf]

Supplemental Figure S1

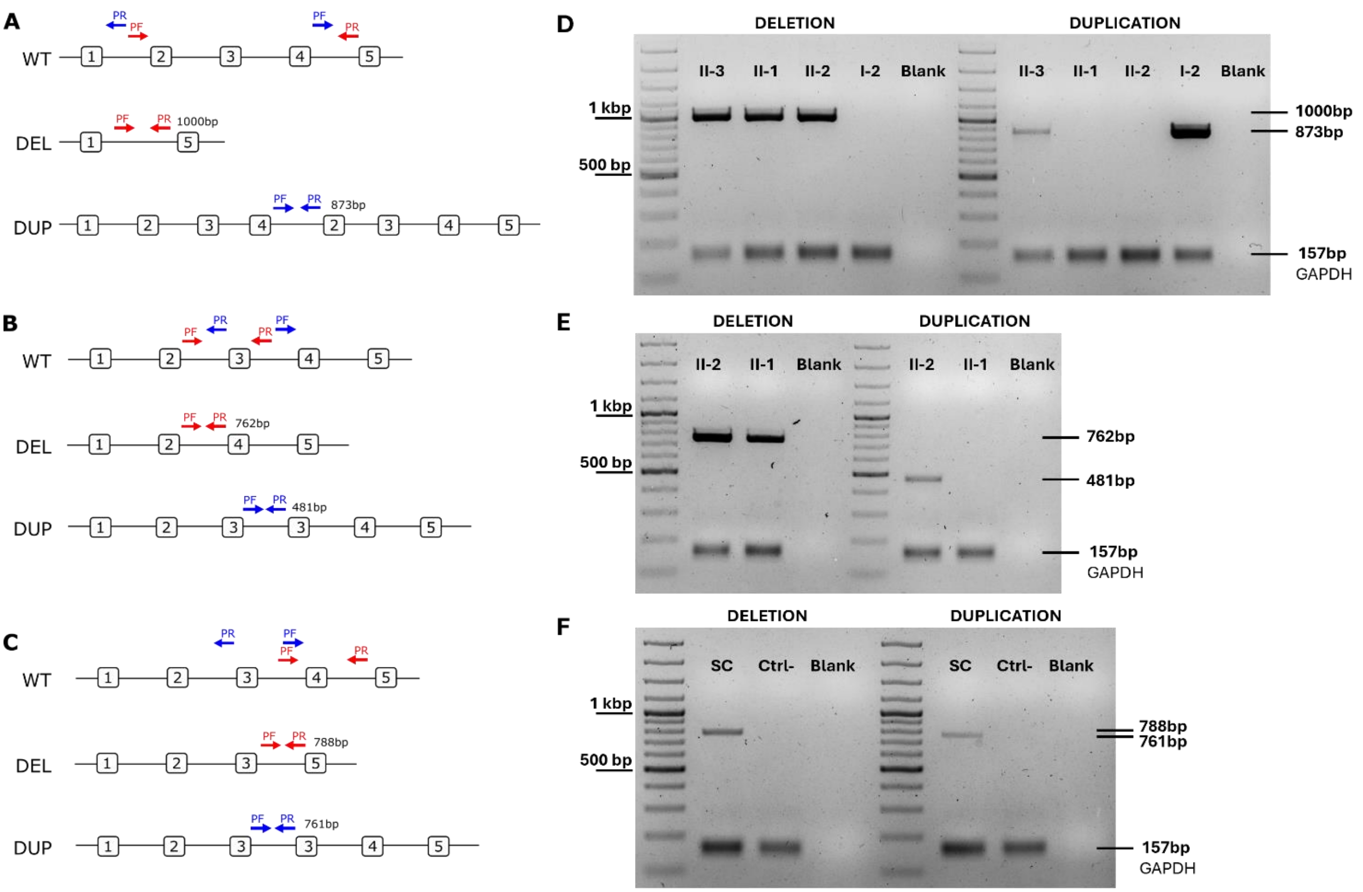

Supplemental Fig. S1. Validation of the identified compound heterozygous structural variations of *PRKN* by genomic PCR analysis. Primers designed to detect deletions (blue) and duplications (red) in family A (**A**), family B (**B**), and the single case (**C**). Agarose gel electrophoresis of the PCR products around the breakpoints of the deletion (left part of the gel) and the duplication (right part of the gel) in individuals II-3, II-1, II-2 and I-2 of family A (**D**), in individuals II-2 and II-1 of family B (**E**), and in the single case (**F**). Amplicon sizes (bp) are indicated; amplicon size for GAPDH control is 157bp.

PF, primer forward; PR, primer reverse; WT, wild-type; DEL, deletion; DUP, duplication; Ctrl-, negative control; SC, single case.
