# Supplementary material for: Compound Heterozygous Structural Variants in Cases with Unsolved PRKN ‐Associated Parkinson's Disease
Source: Mov Disord. 2025 Aug 30;40(12):2722–31. doi: 10.1002/mds.70027 (PMC12710201; doi:10.1002/mds.70027)
Supplement: Supplementary file 2 — Fig. S2. Multiple ligation‐dependent probe amplification (MLPA) analysis of a panel of Parkinson's disease (PD)‐associated genes, including PRKN. Representative images of MLPA analysis for one PD affected member of family A (A), of family B (B), and the single case (C). [file MDS-40-2722-s002.pdf]

Supplemental Figure S2

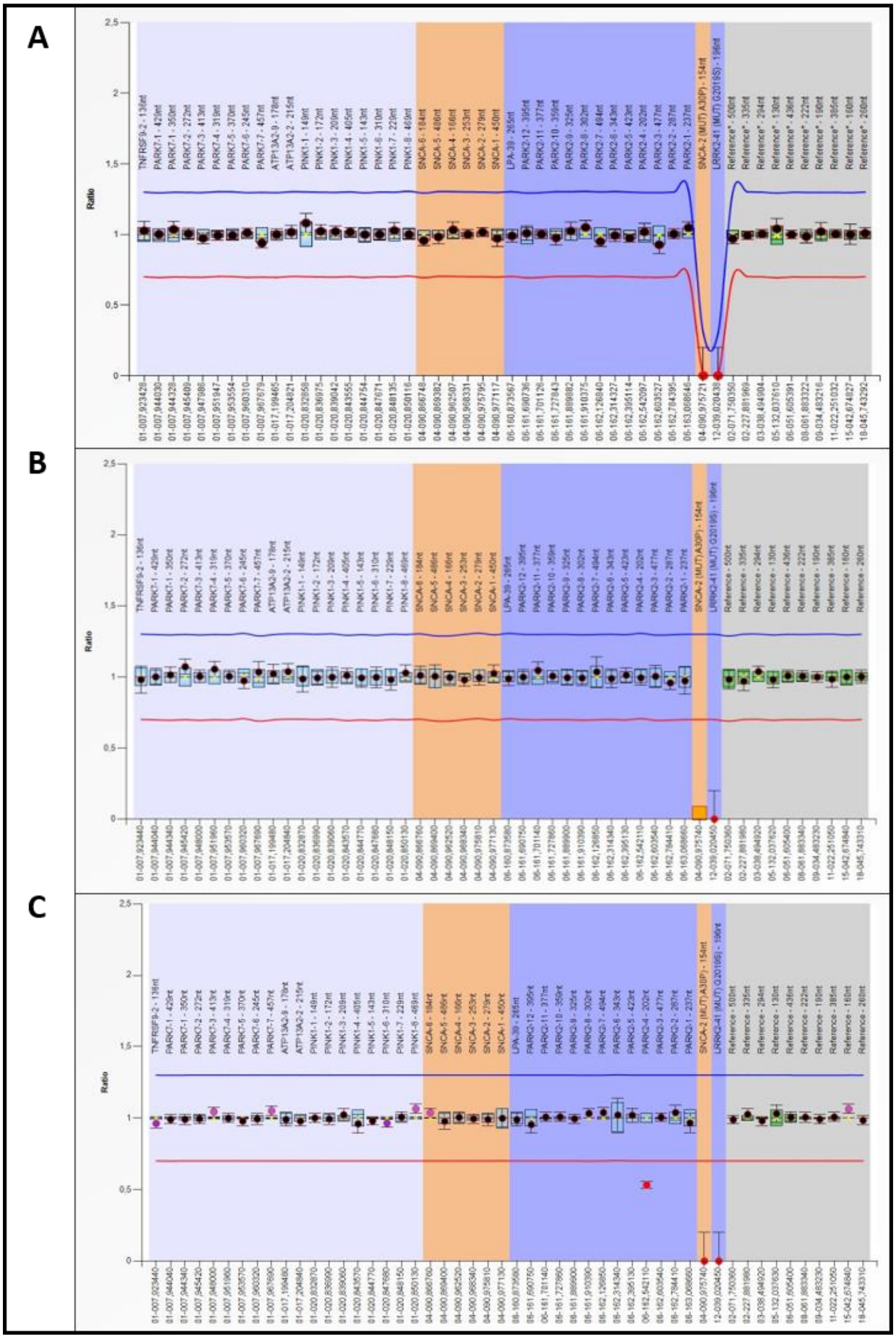

Supplemental Fig. S2. Multiple Ligation-dependent Probe Amplification (MLPA) analysis of a panel of PD-associated genes, including *PRKN*. Representative images of MLPA analysis for one PD affected member of family A (**A**), of family B (**B**) and single case (**C**).
