# Supplementary material for: Compound Heterozygous Structural Variants in Cases with Unsolved PRKN ‐Associated Parkinson's Disease
Source: Mov Disord. 2025 Aug 30;40(12):2722–31. doi: 10.1002/mds.70027 (PMC12710201; doi:10.1002/mds.70027)
Supplement: Supplementary file 3 — Fig. S3. Analysis of the identified biallelic PRKN structural variants by long‐read sequencing in family B and the single case. Representative images from the Integrative Genomics Viewer (IGV) of long‐read sequencing split reads mapped on the PRKN gene showing both the deletion (blue) and the duplication (red) of exon 3 in the two affected siblings (A) and only the deletion (blue) of exon 3 in the healthy brother (B) of family B, and with the concomitant deletion (blue) of exon 3 and 4 and duplication (red) of exon 3 in the single case (C). Gray reads indicate default state for reads that match the reference genome. Pink and violet reads are supplementary alignments. [file MDS-40-2722-s008.pdf]

Supplemental Figure S3

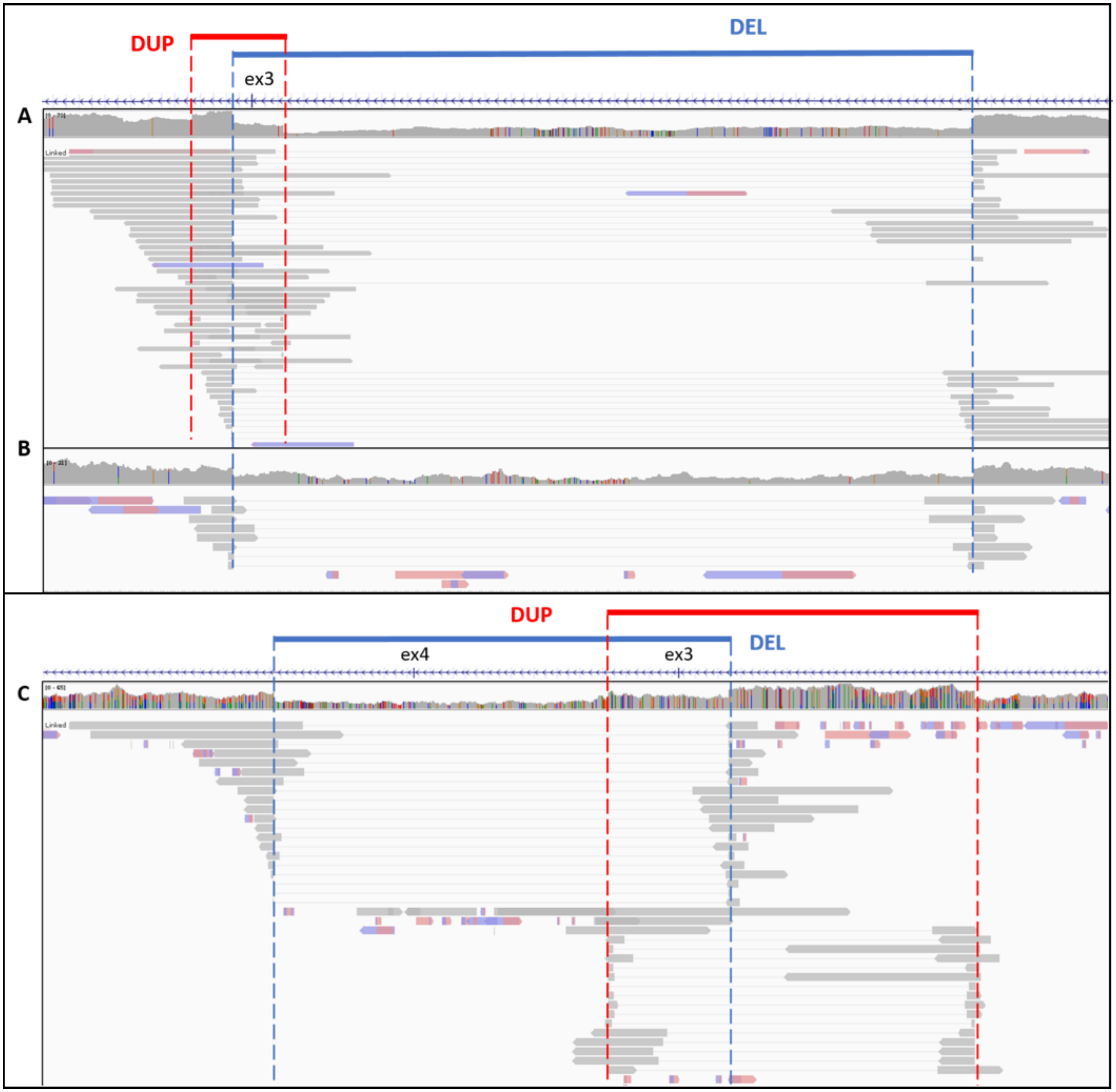

Supplemental Fig. S3. Analysis of the identified biallelic *PRKN* structural variants by long-read sequencing in family B and the single case. Representative images from the Integrative Genomics Viewer (IGV) of long-read sequencing split reads mapped on the *PRKN* gene showing both the deletion (blue) and the duplication (red) of exon 3 in the two affected siblings (**A**) and only the deletion (blue) of exon 3 in the healthy brother (**B**) of family B, and with the concomitant deletion (blue) of exon 3 and 4 and duplication (red) of exon 3 in the single case (**C**). Gray reads indicate default state for reads that match the reference genome. Pink and violet reads are supplementary alignments.
