# Supplementary material for: Compound Heterozygous Structural Variants in Cases with Unsolved PRKN ‐Associated Parkinson's Disease
Source: Mov Disord. 2025 Aug 30;40(12):2722–31. doi: 10.1002/mds.70027 (PMC12710201; doi:10.1002/mds.70027)
Supplement: Supplementary file 4 — Table S1. Clinical and demographic characteristics of patients included in the study. The table summarizes sex, age at disease onset, ethnicity, and family history of the disease. [file MDS-40-2722-s005.pdf]

Supplemental Table S1. Clinical and demographic characteristics of patients included in the study. The table summarizes sex, age at disease onset, ethnicity, and family history of the disease.

		**EOPD (n=498)**
Sex	M	304
	F	194
Mean onset (±SD)		42.53 (± 6.28)
Race	EUR	482
	AMR	7
	AFR	4
	SAS	3
	EAS	2
Family History	Yes	69
	No	179
	NA	250

Abbreviations: AFR, African; AMR, Ad Mixed American; EAS, East Asian; EUR, European; SAS, South Asian
