# Supplementary material for: Compound Heterozygous Structural Variants in Cases with Unsolved PRKN ‐Associated Parkinson's Disease
Source: Mov Disord. 2025 Aug 30;40(12):2722–31. doi: 10.1002/mds.70027 (PMC12710201; doi:10.1002/mds.70027)
Supplement: Supplementary file 5 — Table S2. Summary metrics of short‐read whole genome sequencing (sr‐WGS) data of the two family members and sporadic Parkinson's disease (PD) patient with compound heterozygous PRKN structural variants (SVs). [file MDS-40-2722-s006.pdf]

Supplemental Table S2. Summary metrics of sr-WGS data of the two family members and sporadic PD patient with compound heterozygous *PRKN* SVs

Family	Subject	Length (bp)	% Dup	% GC	G bp	Cov(X)
A	I-1	148	27%	41%	110.43	33.5
	I-2	148	28%	41%	125.31	38.0
	II-1	148	25%	41%	90.54	27.4
	II-2	148	28%	41%	123.63	37.5
	II-3	148	28%	41%	153.96	46.7
	II-4	148	26%	41%	98.46	29.8
	II-5	148	28%	41%	116.76	35.4
B	II-1	148	28%	41%	153.81	46.6
	II-2	148	31%	41%	189.03	57.3
	II-3	148	24%	41%	113.91	34.5
Single case	SC	148	33%	41%	141.75	43.0

Abbreviations: % Dup, percentage of Duplicate Reads; GC, content in GC; G bp, Giga base pairs; Cov(X), coverage.
