# Supplementary material for: Compound Heterozygous Structural Variants in Cases with Unsolved PRKN ‐Associated Parkinson's Disease
Source: Mov Disord. 2025 Aug 30;40(12):2722–31. doi: 10.1002/mds.70027 (PMC12710201; doi:10.1002/mds.70027)
Supplement: Supplementary file 6 — Table S3. Summary metrics of long‐read whole genome sequencing (lr‐WGS) data of the two family members and the single case with compound heterozygous PRKN structural variants (SVs). [file MDS-40-2722-s001.pdf]

Supplemental Table S3. Summary metrics of lr-WGS data of the two family members and the single case with compound heterozygous *PRKN* SVs

Family	Subject	Reads (M)	G bp	Cov (X)	N50 (kb)	DEL (ref,alt)	DUP (ref,alt)
A	I-1	4,86	69,70	21.1	24.49	15,19	/
	I-2	5,98	90,96	27.6	25.83	/	19,9
	II-1	6,26	80,4	24.4	22.70	7,9	/
	II-2	3,84	62,00	18.8	26.80	6,10	/
	II-3	4,86	53,58	16.2	24.19	14,5	14,10
	II-4	6,53	100,51	30.5	25.32	26,13	22,9
	II-5	3,44	57,48	17.4	26.76	8,10	6,7
B	II-1	9,77	63,15	19.1	9.18	7,8	/
	II-2	3,72	59,39	18.0	26.98	12,11	10,6
	II-3	9,6	125,27	38.0	22.42	27,29	19,19
Single case	SC	19,68	94,25	28.6	13.08	18,19	17,16

Abbreviations: M, millions; G bp, Giga base pairs; Cov(X), coverage; N50, length of the shortest read required to cover at least 50% of the total sequence length, DEL (ref,alt), reads supporting reference and alternative allele for deletion; DUP (ref,alt), reads supporting reference and alternative allele for duplication.
