# Supplementary material for: Compound Heterozygous Structural Variants in Cases with Unsolved PRKN ‐Associated Parkinson's Disease
Source: Mov Disord. 2025 Aug 30;40(12):2722–31. doi: 10.1002/mds.70027 (PMC12710201; doi:10.1002/mds.70027)
Supplement: Supplementary file 7 — Table S4. Primer sequences designed around the junction breakpoints for polymerase chain reaction (PCR) analysis. [file MDS-40-2722-s004.pdf]

Supplemental Table S4 Primer sequences designed around the junction breakpoints for PCR analysis

PRKN SV TYPE	FWD Primer	Melting Temperature (°C)	REV Primer	Melting Temperature (°C)	Product length (bp)
DEL family A	ACTCCTCAGTTACGGCTCAAC	59.73	CCTCTGACATATGACCGCCC	59.97	1000
DUP family A	TGCCGCTGACACATCCTTT	59.93	GCCTGGATGTTCTGTGTCGG	61.02	873
DEL family B	TCTTACCACAATACCATGTTGAGT	57.88	AGTTCTGAAAAGGATTCCCTCCA	59.34	762
DUP family B	GGGGAAAGGTGGACCAAGAA	59.52	CACCTCTCTGTGCTCACGAC	60.39	481
DEL single case	ATCTCCTGGCTCCAAAGCAC	60.03	CTGTGTCTGCGTCTGACCAT	60.04	788
DUP single case	GAATGACGGAGGCATGTCGT	59.89	CTGAGCTAGCAGCAGGACAA	60.11	761

Abbreviations: SV, structural variant; DEL, deletion; DUP, duplication; FWD, forward primer; REV, reverse primer.
