# Supplementary material for: Compound Heterozygous Structural Variants in Cases with Unsolved PRKN ‐Associated Parkinson's Disease
Source: Mov Disord. 2025 Aug 30;40(12):2722–31. doi: 10.1002/mds.70027 (PMC12710201; doi:10.1002/mds.70027)
Supplement: Supplementary file 8 — Table S5. Clinical and demographic characteristics of Parkinson's Progression Markers Initiative (PPMI) patients included in the study. The table provides information on sex, age at disease onset, ethnicity, age at diagnosis, family history of the disease, and the presence of key motor symptoms. [file MDS-40-2722-s009.pdf]

Supplemental Table S5. Clinical and demographic characteristics of PPMI patients included in the study. The table provides information on sex, age at disease onset, ethnicity, age at diagnosis, family history of the disease, and the presence of key motor symptoms.

		**PD-NA**	**EOPD (<=50)**	**LOPD**	**Prodromal**	**SWEDD**	**HC**
N subjects	TOT=851	8	87	354	144	63	195
Sex	Male	3	52	237	80	39	126
	Female	5	35	117	64	24	69
Mean onset (±SD)		/	44.42 (±5.51)	63.32 (±6.84)	58.30 n=1*	58.69 (±10.77) n=60*	/
Race	White	8	80	333	140	60	179
	Black	0	1	5	2	1	10
	Asian	0	2	6	0	1	1
	Other (includes multi-racial)	0	3	10	1	1	5
Age at diagnosis (±SD)		63.95 (±6.8)	46.48 (±5.18)	64.5 (±6.87)	58.94 n=1*	60.28(±10.29)	/
Family history	1st Degree Family w/PD	/	11	51	70	15	/
	Non-1st Degree Family w/PD	1	14	37	5	6	10
	No Family w/PD	7	62	266	69	42	185
Tremor	No	1	24	70	/	10	/
	Yes	7	63	284	1	52	/
	NA	/	/	/	143	1	/
Rigidity	No	2	14	94	/	26	/
	Yes	6	72	256	1	36	/
	NA	/	1	4	143	1	/
Bradycinesia	No	/	14	69	/	12	/
	Yes	8	72	283	1	50	/
	NA	/	1	2	143	1	/
Postural Instability	No	7	79	323	1	54	/
	Yes	1	6	27	/	8	/
	NA	/	2	4	143	1	/

Abbreviations: PD-NA, PD patients with no onset available; EOPD, early-onset PD; LOPD, late-onset PD; Prodromal, Participants who are at risk of Parkinson's based on clinical features, genetic variants, or other biomarkers; SWEDD, patients clinically diagnosed with PD with normal DAT SPECT; HC, healthy controls. * Number of subjects with available information on the age of onset.
