# Supplementary material for: Compound Heterozygous Structural Variants in Cases with Unsolved PRKN ‐Associated Parkinson's Disease
Source: Mov Disord. 2025 Aug 30;40(12):2722–31. doi: 10.1002/mds.70027 (PMC12710201; doi:10.1002/mds.70027)
Supplement: Supplementary file 9 — Table S6. Deletions and duplications in PRKN identified in the Parkinson's Progression Markers Initiative (PPMI). [file MDS-40-2722-s007.pdf]

Supplemental Table S6. Deletions and duplications in *PRKN* identified in the Parkinson's progression markers initiative (PPMI)

SV_length	SV_type	Samples_ID	Cohort	Onset	Max_AF	GT	Location
-172265	DEL	PPMISI3050	SWEED	NA	0.00221	0/1	intron1-intron2
-454797	DEL	PPMISI3131	PDsporadic	LOPD	0.00031	0/1	intron3-intron7
148081	DUP	PPMISI3156	HC	NA	0.00031	0/1	intron1-intron2
375430	DUP	PPMISI3160	HC	NA	0.00031	0/1	intron1-intron2
-143358	DEL	PPMISI3185	PDsporadic	LOPD	0.00062	0/1	intron2-intron4
-64839	DEL	PPMISI3268	PDsporadic	LOPD	0.00000	0/1	intron4-intron5
260346	DUP	PPMISI3357	HC	NA	0.00055	0/1	intron1-intron2
198651	DUP	PPMISI3609	PDsporadic	LOPD	0.00055	0/1	intron1-intron2
-174546	DEL	PPMISI4070	PDsporadic	LOPD	0.00005	0/1	intron3-intron5
-236473	DEL	PPMISI40757	PDgenetic	LOPD	0.00047	0/1	intron1-intron2
129117	DUP	PPMISI40779	PDgenetic	EOPD	0.00018	0/1	intron3-intron4
198651	DUP	PPMISI50485	PDgenetic	LOPD	0.00055	0/1	intron1-intron2
198651	DUP	PPMISI50670	Prodromal	NA	0.00055	0/1	intron1-intron2

Abbreviations: SV, structural variant; DEL, deletion; DUP, duplication; SWEED, patients clinically diagnosed with PD with normal DAT SPECT; PDsporadic, patients with untreated PD; PDgenetic, PD patients with pathogenic genetic variant(s) in *LRRK2*, *GBA*, and *SNCA*; HC, participants with no neurologic disorder and no first-degree relative with PD; Prodromal, participants who are at risk of Parkinson's based on clinical features, genetic variants, or other biomarkers; LOPD, late-onset Parkinson's disease; EOPD, early-onset Parkinson's disease; Max_AF, maximum allelic fraction from all matching SVs across all gnomAD, CCDG, and 1000G databases.
